# Erratum: “Causal Contributions of the Domain-General (Multiple Demand) and the Language-Selective Brain Networks to Perceptual and Semantic Challenges in Speech Comprehension”

**DOI:** 10.1162/nol_x_00103

**Published:** 2023-02-17

**Authors:** Lucy J. MacGregor, Rebecca A. Gilbert, Zuzanna Balewski, Daniel J. Mitchell, Sharon W. Erzinçlioğlu, Jennifer M. Rodd, John Duncan, Evelina Fedorenko, Matthew H. Davis

**Affiliations:** MRC Cognition and Brain Sciences Unit, University of Cambridge, Cambridge, UK; Helen Wills Neuroscience Institute, University of California, Berkeley, Berkeley, CA; Psychology and Language Sciences, University College London, London, UK; Department of Brain and Cognitive Sciences, Massachusetts Institute of Technology, Cambridge, MA; McGovern Institute for Brain Research, Massachusetts Institute of Technology, Cambridge, MA; Program in Speech and Hearing Bioscience and Technology, Harvard University, Cambridge, MA

After publication of our paper “Causal Contributions of the Domain-General (Multiple Demand) and the Language-Selective Brain Networks to Perceptual and Semantic Challenges in Speech Comprehension” (https://doi.org/10.1162/nol_a_00081), we discovered an error in Table 1. Specifically, in the two right-most columns, values with more than one digit are missing the first digit. Please see the corrected table below.

**Table 1.** Participant profiles, including initial group assignment, demographics, lesion details, IQ based on the NART, and TROG2 scores.

Participant	Group	Age at test	Sex	Lesion aetiology	Lesion location	Premorbid IQ (from NART)	TROG2 Score	Total lesion volume (cm^3^)
lang1	LANG	50	F	Tumour	L posterior temporal, posterior corpus callosum, posterior cingulum	108	14	29.49
lang2	LANG	75	F	Stroke (ischaemic)	L frontal and anterior insular cortex	86	8	64.06
lang3	LANG	58	F	Stroke (haemorrhagic)	R frontotemporoparietal and anterior thalamus	123	18	139.70
lang4	LANG	65	F	Tumour	L temporal	117	16	21.90
lang5	LANG	50	M	Other (abscess removal)	L temporal	101	14	19.71
lang6	LANG	56	M	Tumour	R inferior parietal/temporal	112	12	42.38
md1	MD	58	M	Stroke (ischaemic)	L occipitoparietal	97	15	78.21
md2	MD	63	F	Tumour	L superior parietal lobe	123	17	37.06
md3	MD	64	M	Other (unknown)	R frontotemporoparietal surrounding insular cortex. Anterior branch of internal capsule	115	15	68.59
md4	MD	53	F	Tumour	R superior frontal	106	14	114.68
md5	MD	69	M	Stroke (haemorrhagic)	L frontal	101	4	113.15
md6^a^	MD	61	M	Tumour + Stroke (haemorrhagic)	R posterior frontal and some anterior/medial, extending to post-central gyrus/parietal areas	123	13	131.27
md7	MD	52	F	Tumour	Bifrontal	112	16	59.02
md8	MD	74	M	Tumour	R frontal	120	16	27.68
md9	MD	67	M	Tumour	L anterior frontal	126	18	24.05
md10^b^	MD	81	M	Stroke (haemorrhagic)	L temporooccipital	81	14	13.26
other1	OTHER	58	M	Other (resection for epilepsy)	R temporal	NA	16	17.84
other2	OTHER	60	M	Stroke (haemorrhagic)	R basal ganglia (putamen + caudate + thalamus) and internal capsule dorsal anterior insula	97	17	25.37
other3	OTHER	37	F	Stroke (haemorrhagic)	L frontal	105	15	12.52

*Note*. NART = National Adult Reading Test (Nelson, 1982); TROG2 = Test of Reception of Grammar (Bishop, 2003); lang/LANG = language; md/MD = multiple demand; L = left; R = right.

^a^ Participant md6 was excluded from the degraded speech task analyses.

^b^ Participant md10 was excluded from the semantic ambiguity task analyses.

The authors confirm that there are no mistakes in the analysis; the error is in the values presented in the table and occurred after acceptance.

